# Age-related radiographic parameters difference between the degenerative lumbar spinal stenosis patients and healthy people and correlation analysis

**DOI:** 10.1186/s13018-022-03374-0

**Published:** 2022-11-03

**Authors:** Donglai Li, Lianlei Wang, Zheng Wang, Chao Li, Suomao Yuan, Yonghao Tian, Xuguang Yu, Xinyu Liu

**Affiliations:** 1 Department of Orthopedics, Qilu Hospital of Shandong University, Jinan, Shandong People’s Republic of China; 2 School of Physical Education, Shandong University, Jinan, Shandong People’s Republic of China

**Keywords:** Degenerative lumbar spinal stenosis, Intervertebral disk height, Cartilaginous endplate failure, Intervertebral disk degeneration, Ligamentum flavum

## Abstract

**Objectives:**

To identify age-related radiographic risk factors for degenerative lumbar spinal stenosis (DLSS) and analyze correlations among them.

**Methods:**

A total of 180 cases were enrolled in this study, and lumbar magnetic resonance was performed. Among them, 93 cases suffered DLSS and lumbar dynamic X-ray was examined. And following parameters were measured and evaluated: intervertebral disk height (IDH), the ratio of IDH(IDH_L4-5/L3-4_), initial IDH of L4-5(iIDH_L4-5_) in the DLSS group, disk degeneration (DD), cartilaginous endplate failure (CEF), Modic changes, the thickness of ligamentum flavum (LF), range of intervertebral motion (ROM), facet joint opening (FJO), facet joint angle (FJA), the standard cross-sectional area (SCSA) of the multifidus, erector spinae, and psoas major muscles. The data of two groups were compared, and the possible risk factors of DLSS were analyzed.

**Results:**

Compared with the control group, the DLSS group had higher IDH except for L4-5 and larger iIDH_L4-5_ (*P* < 0.05). Significant differences were shown in CEF and the thickness of LF at L1-S1 and DD at L4-5 (*P* < 0.05). The DLSS group had smaller SCSA of multifidus, erector spinae, and psoas major muscles but greater FJA, FJO (*P* < 0.05). And the risk of DLSS increased when iIDH_L4-5_ ≥ 10.73 mm, FJA ≥ 52.03° , or FJO ≥ 3.75 mm. IDH positively correlated with SCSA of multifidus and psoas major muscles and ROM at L1-S1 (*P* < 0.05). DD showed negative linear relations with SCSA of multifidus and psoas muscle and positive linear relation with CEF at L1-2, L2-3, and L5-S1 (*P* < 0.05).

**Conclusion:**

Larger initial disk height and excessive CEF may induce DLSS by increasing intervertebral mobility to promote DD, and atrophied paravertebral muscles by weakening the stability of lumbar spine.

## Introduction

Degenerative lumbar spinal stenosis (DLSS) is the vital reason for spinal surgery in patients over 65 years, characterized by a reduction in the volume of the spinal canal and compression of the dural sac and nerve roots [1]. Many degenerative changes happen in the narrow spinal segment, and disk degeneration (DD) usually plays an essential role in the progress, resulting in intervertebral disk height (IDH) decreasing, facet joint hypertrophy, and ligamentum flavum (LF) thickening as a consequence [2].

Normal IDH could prevent the ligamentum flavum from excessively shrinking and disk from bulging and keep sufficient space for intervertebral foramen, and thus the spine canal has enough volume. Previous studies have found that the IDH of DLSS patients decreased, and thus a series of changes happened as a consequence [2]. Ehud et al. [3] found the loss of IDH might lead to facet joint hypertrophy and ligamentum flavum thickening. Cartilaginous endplate (CEP), a kind of fibrous cartilage, maintains the integrity of the disk and overtakes the function of nutrient delivery and metabolite drainage [4]. Rajasekaran et al. [5] suggested that cartilaginous endplate failure (CEF) is an initiating factor for DD, and a previous study found CEF is associated with lumbar disk herniation [6]. Also, the low back pain in patients with DLSS shows a strong link with the Modic changes [7]. However, to our best knowledge, there is no comparative study about CEF and Modic changes in patients with DLSS versus healthy individuals.

As known, disk degenerative progress can also be accelerated by fat infiltration and atrophy of the paravertebral muscles. Nevertheless, the relation between IDH and paravertebral muscles has not been investigated [8]. In addition, the facet joints' anatomical abnormalities might also be a risk factor for DLSS by accelerating the development of DD [9, 10]. Patients with DLSS have more pronounced osteophytes, larger facet joint opening (FJO), and greater facet joint angle (FJA), which means more sagittalized alignment of the facet joints and lumbar instability [9, 10]. And LF thickening is another pathogenic factor for DLSS [11], but whether the thickness of LF in patients with DLSS differs from that in healthy individuals at non-responsible segments is unclear.

Some studies reported that the degeneration of disks, facet joints, and LF were of great magnitude. However, most of them just focused on a single point in the development of DLSS while their interactions and mechanisms in the process of DLSS have not been deeply studied. And few literatures reported the difference in IDH and CEF between DLSS patients and healthy populations. We noticed that and proposed a method to estimate the initial IDH of duty segment of DLSS patients to explain how various elements in spine segment motion interact and work in developing DLSS.

This study investigated the radiographic parameters of the patients with DLSS and healthy individuals to: (1) compare IDH, DD, CEF, Modic changes, LF thickness, FJO, FJA, and area of paravertebral muscles between two samples; (2) analyze the radiographic risk factors for DLSS; (3) explore the possible interrelationship among IDH, CEF, and DLSS.

## Materials and methods

### Study design and population

This retrospective study was approved by our institutional ethics committee, and the requirement for informed consent was waived (KYLL-2021(KS)-249). The inclusion criteria for the DLSS group were as follows: (a) diagnosed with L4-5 central canal stenosis, (b) ineffective after strict conservative treatment for more than 6 months, and (c) complete imaging data. And the exclusion criteria were: (a) previous spinal surgery, (b) non-degenerative stenosis, (c) lumbar disk herniation, (d) spondylolisthesis or instability, which showed as translational motion more than 4 mm at L4-5 or as angulation of a motion segment more than 10° on lateral flexion–extension radiographs, (e) lateral recess stenosis or foraminal stenosis, which were excluded by evaluating axial and sagittal images of MRI. The inclusion criteria for the control group were those who underwent radiographic examinations without obvious abnormality and systematic diseases.

### Radiographic measurements

Both groups underwent lumbar magnetic resonance imaging (MRI) examination, and the patients in the DLSS group also underwent dynamic X-ray examination. All radiographic measurements and findings were independently and double-blindly evaluated by two orthopedic spine surgeons. If appeared inconsistent results, another senior orthopedic spine doctor (the corresponding author) would make the final decision.

**Fig. 1:**
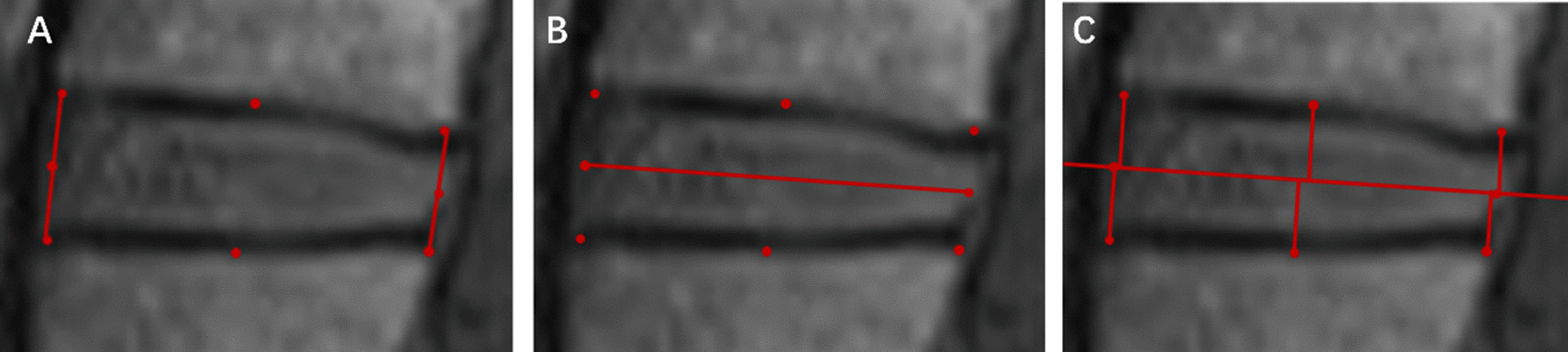
Illustration of the measurement of IDH. **A** Connect the anterior and posterior edges of the adjacent CEPs; **B** the mid-points of the two lines were identified and connected; **C** the perpendicular distances from the anterior, middle, and posterior points on CEP were measured. IDH = intervertebral disk height; CEP = cartilaginous endplate

IDH was measured on the mid-sagittal plane of T1WI according to modified distortion compensated Roentgen analysis [12]. Two longitudinal lines were drawn at the anterior and posterior edges of the disk, and the mid-points of the two lines were identified and connected. The perpendicular distances from the six points on CEP to the mid-line were measured and summed up, and the average value was taken as IDH (Fig. 1). The IDH_L4-5/L3-4_ was the ratio of the IDH of L4-5 and L3-4. Initial intervertebral disk height of L4-5(iIDH_L4-5_) was an estimate derived from the IDH of L3-4 of DLSS patients and averaged IDH_L4-5/L3-4_ in the normal group, which represented the IDH of L4-5 before stenosis occurred in DLSS patients. The iIDH_L4-5_ was calculated as follows:

$${\text{iIDH}}_{{\text{L4 - 5}}} = {\text{IDH}}_{{\text{L3 - 4}}} {\text{ of DLSS group}} \times {\text{IDH}}_{{{\text{L4 - 5}}/{\text{L3 - 4}}}} {\text{ of the control group}}$$

**Fig. 2:**
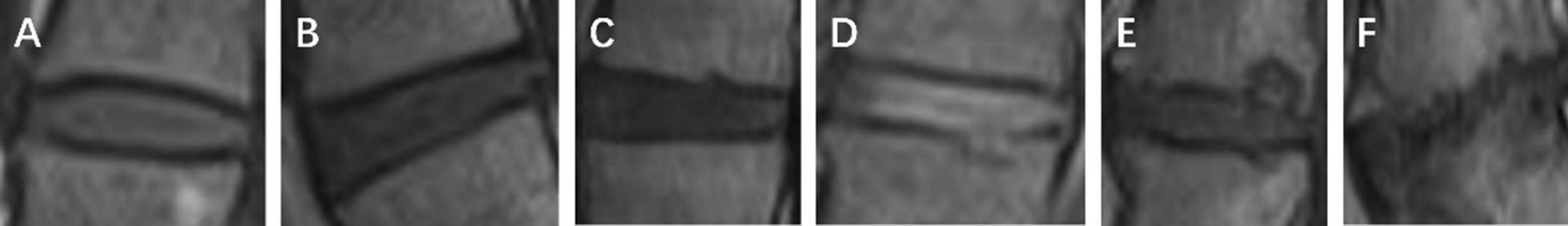
Rajasekaran's classification system for CEP. **A** Grade 1: The structure of CEP is normal, with no crack or defect; **B** Grade 2: CEP is locally thinned without cracks or defects; **C** Grade 3: nucleus pulposus was in contact with bone marrow, but the CEP contour still existed without Modic changes; **D** Grade 4: The defect of CEP reaches 25% and Modic changes usually present; **E** Grade 5: The defect of CEP reaches 50% and Modic changes usually present; **F** Grade 6: CEP is completely damaged and Modic changes usually present. CEP = cartilaginous endplate

DD was assessed by the Pfirrmann grading system [13]. CEF was classified into six grades according to Rajasekaran’s classification [5] on sagittal T1WI (Fig. 2). Grade 1: the structure of CEP is normal without cracks or defects; Grade 2: CEP is locally thinned without cracks or defects; Grade 3: nucleus pulposus was in contact with bone marrow, but the CEP contour still existed without Modic changes; Grade 4: the defect of CEP reaches 25%, and Modic changes usually appear; Grade 5: the defect of CEP reaches 50%, and Modic changes usually appear; and Grade 6: CEP is completely damaged, and Modic changes usually appear. The score of each endplate was equal to its grade (e.g. Grade 3 = 3 points). The total cartilage endplate score (TEP_S_) was derived by adding up the score of both endplates of each disk. Rajasekaran et al. [5] found a certain positively correlation between CEF and DD, so we defined TEPs ≤ 4, 4 < TEPs ≤ 8, and TEPs > 8 as mild, moderate, and severe injury, respectively.

**Fig. 3:**
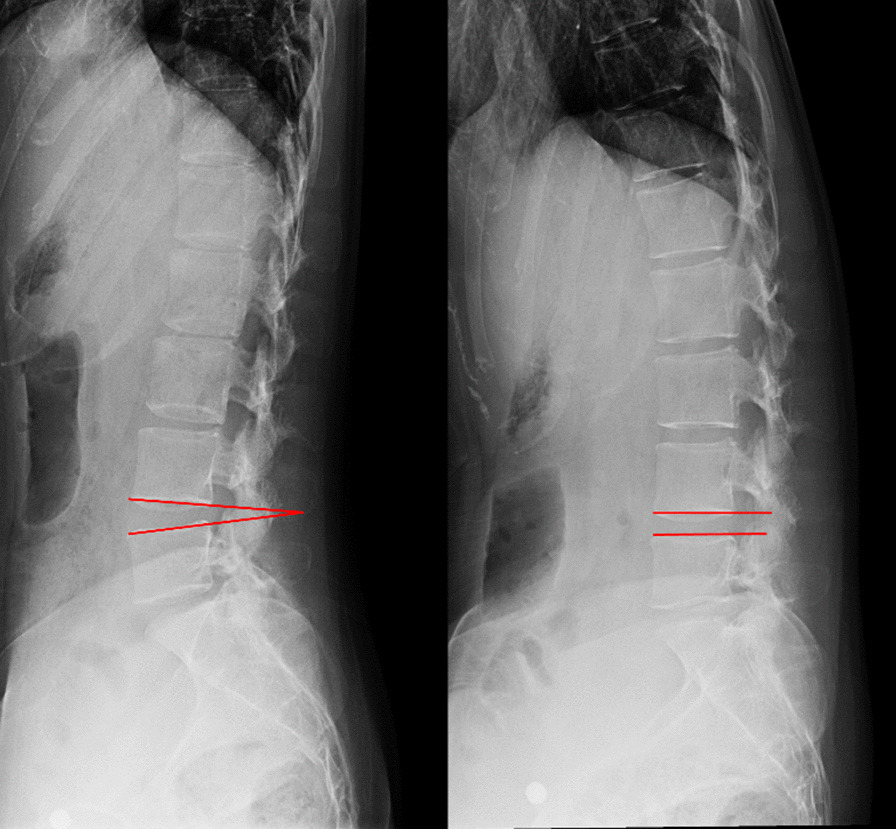
The measurement of intervertebral angle on flexion–extension radiographs

**Fig. 4:**
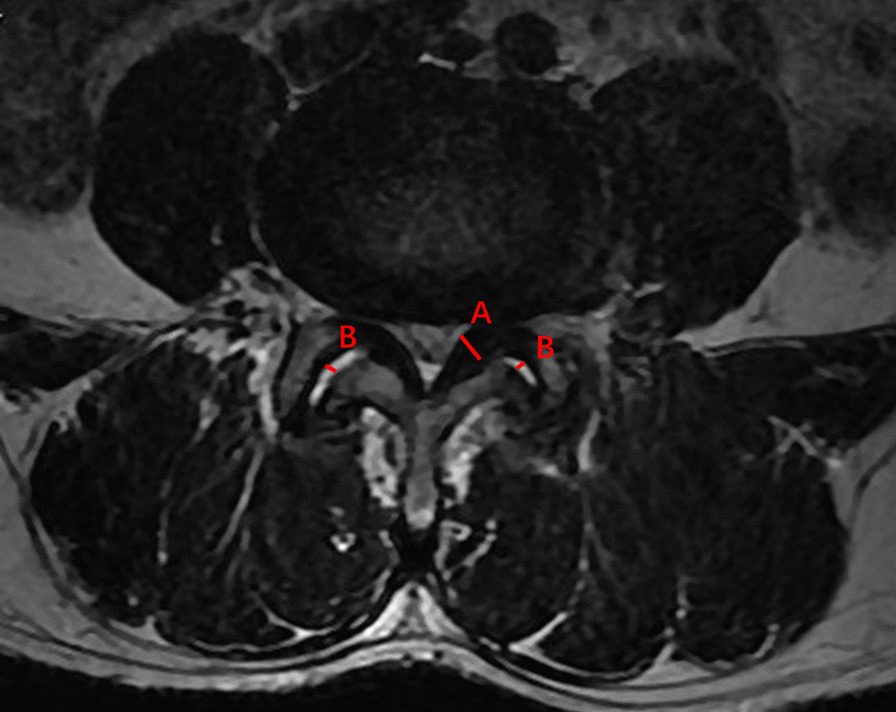
The measurements of DCSA, the thickness of LF, and FJO. **A** LF; **B** FJO. FJO = facet joint opening; LF = ligamentum flavum

**Fig. 5:**
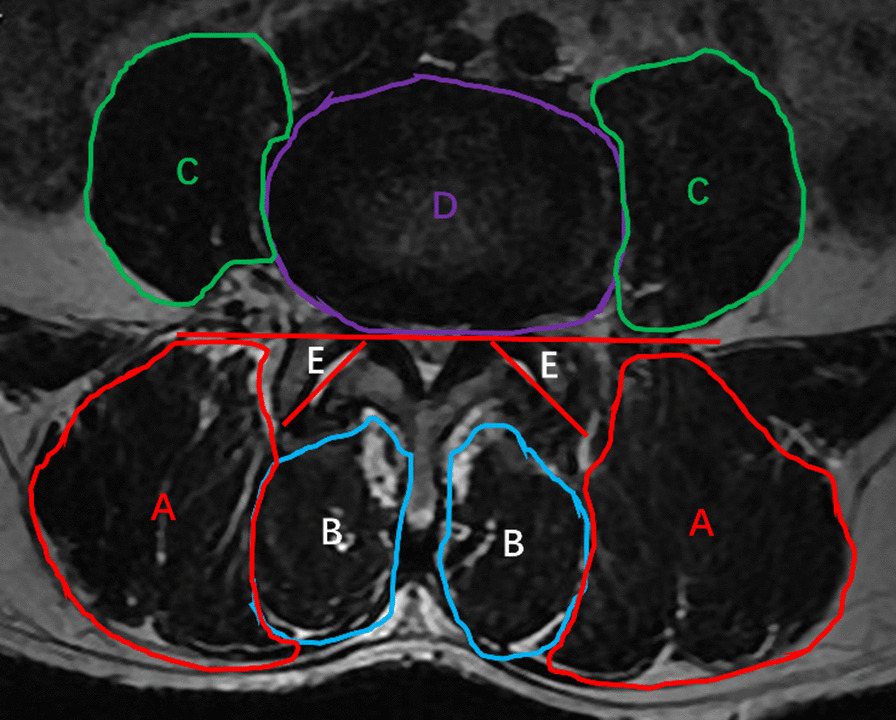
The measurements of the area of paravertebral muscles and FJA. **A** Multifidus muscle; **B** erector spinae muscle; **C** Psoas major muscle. **D** Intervertebral disk; **E** FJA. FJA = facet joint angle

Lumbar intervertebral angles formed with the upper and lower endplates at two ends of L4-5 disks on flexion–extension radiographs were measured, and the corresponding change was defined as ROM (Fig. 3). The anterior and middle 1/3 of the left LF was selected to measure the thickness owing to this part being the thickest [11] (Fig. 4). FJO, FJA, bilateral multifidus, erector spinae, and psoas major muscles were measured at mid-disk level of L4-5. The widest spaces of bilateral facet joints were measured, and the average was considered as FJO (Fig. 5). FJA was measured by averaging the bilateral angles (Fig. 5). The bilateral multifidus, erector spinae, and psoas major muscles were traced with Image J software (version 1.52), and their mean values were calculated and recorded (Fig. 5). Standard cross-sectional area (SCSA) was defined as comparing muscle area and disk area at L4-5 to eliminate individual differences [14].

### Statistical analysis

Pearson’s chi-squared test was used to compare the frequencies of categorical variables. The Mann–Whitney U test was performed for continuous variables, which were presented as the mean ± standard deviation. The inter-rater reliability tests for Modic changes, CEF, and DD were evaluated with kappa coefficient, and IDH, LF, ROM, L4-5 SCSA of paravertebral muscles area, facet joint angle, and opening with Kendall’s concordance coefficient. The correlation analysis was conducted using the parametric Pearson’s or nonparametric Spearman’s correlation coefficients. And receiver operating characteristic (ROC) curve was used to determine the cutoff value. Statistical analyses were performed by using statistical software (SPSS for Windows, version 25.0, IBM, Armonk, NY) and GraphPad Prism (version 7.0, La Jolla, CA).

## Results

A total of 93 patients (31 men, 51–80 years, average 60.00 years ± 10.44) diagnosed with L4-5 DLSS and 87 healthy candidates (25 men, 53–79 years, average 56.67 years ± 9.47) who underwent radiographic examination were enrolled. The baseline information was no statistical difference between the two groups (*P* > 0.05). The Kappa values for two orthopedic spine surgeons in evaluating Modic changes, CEF and DD were 0.835 (*P* < 0.001), 0.820 (*P* < 0.001), and 0.824 (*P* < 0.001), respectively. And Kendall’s concordance coefficients for IDH, LF, ROM, FJA, FJO, and SCSA of the multifidus, erector spinae, and psoas major muscles were 0.846 (*P* < 0.001), 0.822 (*P* < 0.001), 0.821 (*P* < 0.001), 0.828 (*P* < 0.001), 0.934 (*P* < 0.001), 0.876 (*P* < 0.001), 0.853 (*P* < 0.001), and 0.837 (*P* < 0.001), respectively. And good consistency was observed between the two doctors in all radiographic parameters. The radiographic findings are shown in Tables 1, 2, 3, 4, 5, 6, 7 and 8, respectively.

**Table 1:** Comparison of MRI findings between the DLSS group and the control group

	The DLSS group	The control group	*P*
IDH, IDH_L4-5/L3-4_, iIDH_L4-5_			
L1-2	8.65 ± 1.03	6.66 ± 1.39	< 0.001*
L2-3	9.44 ± 0.95	7.49 ± 1.18	< 0.001*
L3-4	9.43 ± 1.34	8.94 ± 1.49	0.021*
L4-5	10.09 ± 1.89	9.81 ± 1.75	0.319
L5-S1	11.08 ± 2.15	8.40 ± 2.07	< 0.001*
IDH L4-5/L3-4	1.08 ± 0.19	1.12 ± 0.23	0.188
iIDH L4-5	10.56 ± 1.49	9.81 ± 1.74	0.002*
The thickness of LF			
L1-2	4.09 ± 0.59	2.86 ± 0.43	< 0.001*
L2-3	4.22 ± 0.56	2.85 ± 0.40	< 0.001*
L3-4	4.64 ± 0.84	3.46 ± 0.62	< 0.001*
L4-5	5.50 ± 1.37	3.79 ± 0.73	< 0.001*
L5-S1	4.21 ± 0.86	2.95 ± 0.49	< 0.001*
ROM			
L1-2	3.23 ± 3.01	–	–
L2-3	4.06 ± 2.62	–	–
L3-4	4.95 ± 3.62	–	–
L4-5	6.11 ± 4.82	–	–
L5-S1	4.70 ± 4.34	–	–
FJA, FJO and the SCSA of paravertebral muscles			
FJA	53.76 ± 10.38	43.46 ± 8.16	< 0.001*
FJO	4.10 ± 0.84	3.54 ± 0.44	< 0.001*
Multifidus muscle	0.42 ± 0.093	0.48 ± 0.12	< 0.001*
Erector spinal muscle	1.16 ± 0.29	1.43 ± 0.34	< 0.001*
Psoas major muscle	1.01 ± 0.31	1.25 ± 0.38	< 0.001*

Values are mean ± SD. *IDH* intervertebral disk height, *IDH*_*L4-5/L3-4*_ the ratio of IDH between L4-5 and L3-4, *iIDH*_*L4-5*_ initial IDH of L4-5 in the DLSS group, *LF* ligamentum flavum, *ROM* range of intervertebral motion, *FJA* facet joint angle, *FJO* facet joint opening, *SCSA* standard cross-sectional area

*P < 0.05

**Table 2:** Comparison of DD between the DLSS group and the control group

	The DLSS group					The control group					*P*
	1	2	3	4	5	1	2	3	4	5	
L1-2	0	29	47	17	0	2	33	40	12	0	0.322
L2-3	0	25	45	21	2	0	28	44	15	0	0.394
L3-4	0	4	43	35	0	0	9	43	35	0	0.102
L4-5	0	1	24	66	2	2	5	42	38	0	0.001*
L5-S1	0	17	27	31	18	0	4	30	43	10	0.07

Values are frequency. *DD* disk degeneration

*P < 0.05

**Table 3:** Comparison of CEF between the DLSS group and the control group

	The DLSS group			The control group			*P*
	Mild	Moderate	Severe	Mild	Moderate	Severe	
L1-2	34	58	1	80	7	0	< 0.001*
L2-3	17	75	1	77	10	0	< 0.001*
L3-4	0	58	35	18	64	5	< 0.001*
L4-5	0	12	81	15	64	8	< 0.001*
L5-S1	13	66	14	56	24	7	< 0.001*

Values are frequency. *CEF* cartilaginous endplate

*P < 0.05

**Table 4:** Comparison of Modic changes between the DLSS group and the control group

	The DLSS group				The control group				*P*
	0	1	2	3	0	1	2	3	
L1-2	90	0	2	1	82	0	3	2	0.703
L2-3	86	0	4	3	86	0	1	0	0.100
L3-4	84	0	7	2	82	0	5	0	0.340
L4-5	75	0	14	4	71	0	12	4	0.969
L5-S1	71	0	20	2	57	0	27	3	0.276

Values are frequency

**Table 5:** The correlation analysis between IDH and DD, CEF, Modic changes, the thickness of LF, ROM

	DD		CEF		Modic changes		The thickness of LF		ROM	
	*r*	*P*	*r*	*P*	*r*	*P*	*r*	*P*	*r*	*P*
L1-2	− 0.187	0.073	− 0.123	0.240	− 0.043	0.681	− 0.027	0.795	0.389	< 0.001*
L2-3	− 0.384	< 0.001*	− 0.307	0.030*	− 0.354	0.800	0.041	0.697	0.316	0.002*
L3-4	− 0.313	0.002*	− 0.098	0.350	− 0.089	0.396	− 0.299	0.235	0.324	0.005*
L4-5	− 0.303	0.007*	− 0.181	0.082	− 0.011	0.920	− 0.068	0.519	0.314	0.002*
L5-S1	− 0.498	< 0.001*	− 0.447	< 0.001*	− 0.140	0.160	0.141	0.179	0.472	< 0.001*

*IDH* intervertebral disk height, *DD* disk degeneration, *LF* ligamentum flavum, *ROM* range of motion

*P < 0.05

**Table 6:** The correlation analysis between IDH and FJA, FJO, the SCSA of paravertebral muscles

	*r*	*P*
FJA	− 0.018	0.862
FJO	− 0.075	0.474
Multifidus muscle	− 0.231	0.026*
Erector spinae muscle	0.325	0.001*
Psoas major muscle	0.270	0.009*

*FJA* facet joint angle, *FJO* facet joint opening, *SCSA* standard cross-sectional area

*P < 0.05

**Table 7:** The correlation analysis among other radiographic parameters

	*r*	*P*
The correlation analysis between DD and other radiographic parameters		
FJA	− 0.161	0.123
FJO	0.108	0.301
Multifidus muscle	− 0.387	< 0.001*
Erector spinae muscle	− 0.274	0.008*
Psoas major muscle	− 0.435	< 0.001*
The correlation analysis between CEF and other radiographic parameters		
FJA	0.150	0.152
FJO	0.128	0.221
Multifidus muscle	− 0.067	0.522
Erector spinae muscle	− 0.073	0.485
Psoas major muscle	0.065	0.536
The correlation analysis between LF and other radiographic parameters		
FJA	− 0.297	0.036*
FJO	0.249	0.016*
Multifidus muscle	0.004	0.972
Erector spinae muscle	0.054	0.605
Psoas major muscle	− 0.233	0.001*
The correlation analysis between FJA and other radiographic parameters		
Multifidus muscle	− 0.170	0.104
Erector spinae muscle	− 0.133	0.204
Psoas major muscle	− 0.075	0.477
ROM	− 0.143	0.177
The correlation analysis between FJO and other radiographic parameters		
Multifidus muscle	− 0.008	0.936
Erector spinae muscle	0.009	0.930
Psoas major muscle	− 0.086	0.414
ROM	− 0.094	0.376
The correlation analysis between ROM and other radiographic parameters		
Multifidus muscle	0.091	0.393
Erector spinae muscle	0.077	0.470
Psoas major muscle	− 0.146	0.168

*DD* disk degeneration, *FJA* facet joint angle, *FJO *facet joint opening, *SCSA* standard cross-sectional area, *CEF* cartilaginous endplate, *LF* ligamentum flavum, *ROM* range of motion

*P < 0.05

**Table 8:** The correlation analysis among other radiographic parameters

	DD and CEF		DD and LF		DD and ROM		CEF and LF		CEF and ROM		LF and ROM	
	*r*	*P*	*r*	*P*	*r*	*P*	*r*	*P*	*R*	*P*	*r*	*P*
L1-2	0.408	< 0.001*	− 0.051	0.627	0.024	0.819	0.007	0.950	− 0.055	0.601	− 0.393	0.101
L2-3	0.421	< 0.001*	0.019	0.859	− 0. 233	0.002*	− 0.014	0.892	− 0.235	0.024*	0.018	0.868
L3-4	0.169	0.106	0.105	0.317	− 0.098	0.348	− 0.083	0.431	− 0.191	0.066	0.072	0.493
L4-5	0.012	0.911	0.204	0.050	0.043	0.686	− 0.014	0.894	0.066	0.536	− 0.142	0.178
L5-S1	0.396	< 0.001*	0.114	0.279	− 0. 243	0.001*	0.163	0.118	− 0.176	0.096	− 0.222	0.034

*DD* disk degeneration, *CEF* cartilaginous endplate, *LF* ligamentum flavum, *ROM* range of motion

*P < 0.05

**Fig. 6:**
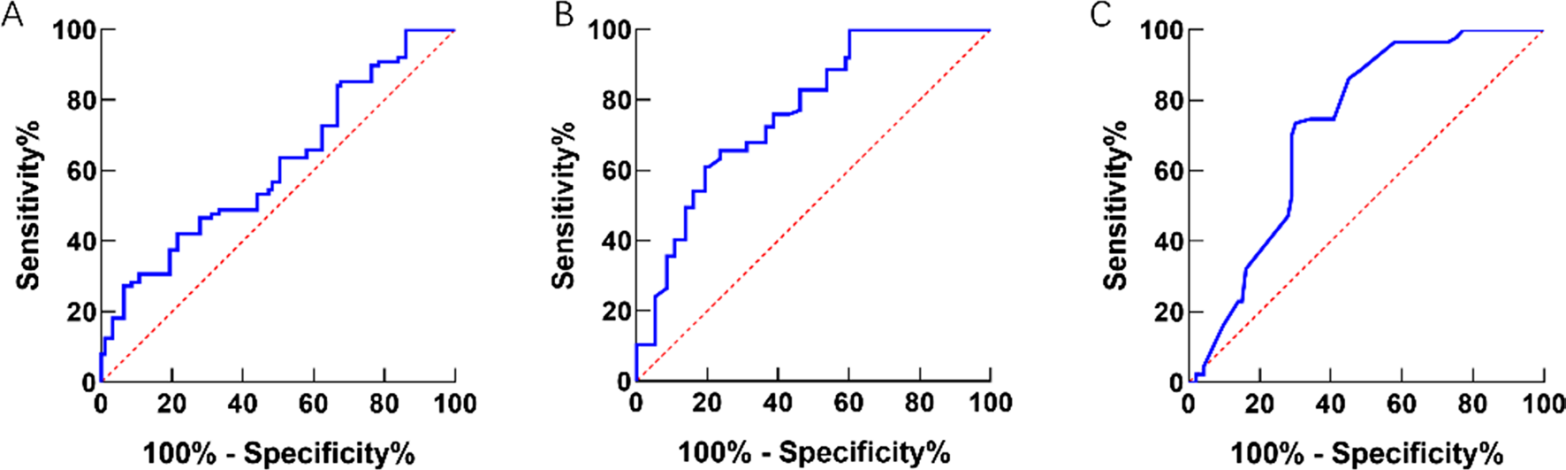
ROC analysis between DLSS and IDH, FJA, FJO. **A** ROC between IDH and DLSS, AUC = 0.772, the cutoff value of IDH = 10.73 mm, sensitivity = 63.46%, specificity = 69.81%; **B** ROC between FJA and DLSS, AUC = 0.776, the cutoff value of FJA = 52.03°, sensitivity = 82.35%, specificity = 60%; **C** ROC between FJO and DLSS, AUC = 0.714, the cutoff value of FJO = 3.75 mm, sensitivity = 73.08%, specificity = 68.63%. IDH = intervertebral disk height; FJA = facet joint angle; FJO = facet joint opening; DLSS = degenerative lumbar spinal stenosis

Compared with the control group, the DLSS group had larger IDH at L1-2, L2-3, L3-4, L5-S1 and higher iIDH_L4-5_ (*P* < 0.05). And also the DLSS group had smaller IDH_L4-5/L3-4_ with no significant difference (*P* = 0.188). The risk for developing DLSS increased when iIDH_L4-5_ ≥ 10.73 mm according to the result of ROC (Fig. 5). The DLSS group had worse CEF and thicker LF at all lumbar segments and more severe DD at L4-5 (*P* < 0.05) (Tables 1, 2, 3). In addition, the DLSS group has smaller SCSA of the multifidus (*P* < 0.001), erector spinae (*P* < 0.001), and psoas major muscles (*P* < 0.001) at L4-5. Lastly, larger FJA and FJO were observed in the DLSS group (*P* < 0.001). The ROC showed that the risk of suffering DLSS was promoted when FJA ≥ 52.03° and/or FJO ≥ 3.75 mm (Fig. 6).

The relationship between IDH and other radiographic parameters in DLSS group is shown in Tables 5, 6, 7 and 8. IDH showed a negative linear relation with DD at L2-3 (*r* = − 0.384, *P* < 0.001), L3-4 (*r* = − 0.313, *P* = 0.002), L4-5 (*r* = − 0.278, *P* = 0.007), L5-S1 (*r* = − 0.498, *P* < 0.001). And negative linear relations appeared between IDH and CEF at L2-3 (*r* = − 0.307, *P* = 0.030), L5-S1 (*r* = − 0.447, *P* < 0.001). There was a significantly positive relation between IDH and ROM (*P* > 0.005), and no association with Modic changes or the thickness of LF (*P* < 0.005) at all lumbar spine. At L4-5, IDH showed a positive relation with the SCSA of the multifidus (*r* = 0.325, *P* = 0.001) and psoas major muscles (*r* = 0.454, *P* < 0.001). DD showed a negative linear relation with the SCSA of the multifidus (*r* = − 0.387, *P* < 0.001) and psoas major muscles (*r* = − 0.435, *P* < 0.001). And DD was correlated with CEF at L1-2 (*r* = 0.408, *P* < 0.001), L2-3 (*r* = 0.421, *P* < 0.001), L5-S1 (*r* = 0.396, P < 0.001), while no relation was observed at L3-4, L4-5.

## Discussion

Previous studies have investigated the correlation between IDH and other radiographic parameters on the diseased segment [1], while the features of the non-diseased segment were not deeply researched. So, both diseased and non-diseased segments were measured in our study aiming to clarify the overall radiographic differences between the DLSS and healthy individuals.

This paper found that IDH in the DLSS group was significantly higher than that in the control group at all lumbar segments except for L4-5, suggesting IDH in DLSS patients may be higher. Also, IDH_L4-5/L3-4_ was smaller in the DLSS group than in the control group. Although a significant difference was not observed in this parameter, it still suggested a decrease in IDH at the responsible segment in the DLSS group. As a result, iIDH_L4-5_ in DLSS group was significantly higher than the IDH of L4-5 in the control group. Bai et al. [15] reported that IDH_L4-5/L3-4_ in Chinese population was 1.14, and the ratio we calculated in the healthy individuals was 1.12 ± 0.23, which was extremely close to the reported value. So, the iIDH_L4-5_ we estimated was reliable and it could represent the initial IDH of DLSS patients in this study when they didn’t suffer from DLSS. Anna et al. [16] found that higher intervertebral disks are more prone to degeneration after undergoing greater deformation and stress during extension and rotation movements, and IDH would decrease by 0.98–1.6 mm if DD increased by one level while the non-DLSS individuals with lower disks had smaller intervertebral motion, and disks could keep their height. Even though, the IDH in the Control group was still lower, for the initial disk height of DLSS patients was exaggeratively high.

Vergroesen et al. [17] proposed a vicious circle that the disk begins to degenerate due to long-term excessive stress, enhanced catabolism of nucleus pulposus cells, and disruption of the extracellular matrix structure. Based on the theory, higher disks bear more stress and are easier to enter the vicious circle and IDH begins to lose as a consequence, which is consistent with our conclusion. Furthermore, the injury and degeneration at L3-4 and L4-5 are more severe, because the segments are located at a transition area from the rigid sacrum to the active lumbar spine with the largest motion of the intervertebral space, whereas the intervertebral spaces of L1-2 and L2-3 have relatively less mobility and L5-S1 is below the posterior superior iliac spine with the protection of transverse process and strong ligament [16, 18]. As a result, a significant difference in DD was observed at L4-5 between the DLSS group and the control group. However, DD showed no significant relation with ROM, probably due to the lateral and shear stresses causing a greater impact on the intervertebral disk [16].

Maxim et al. [19] found that mechanical stress caused early degeneration of the intervertebral disk as well as the facet joint. Conversely, the degenerative facet joints lead to abnormal stress and accelerate the degeneration of the intervertebral disk. Interestingly, Liu et al. [20] compared the FJA of lumbar spondylolisthesis and healthy population and found the facet joints of spondylolisthesis patients are more sagittalized. The sagittalized facet joints have less ability to limit the mobility of spine motion [21] and lumbar spondylolisthesis might occur. And it may be also a risk factor for DLSS. This study found that the risk for developing DLSS increased when FJA ≥ 52.03°. Hasegawa et al. [10] reported the volume of the facet joint is associated with lumbar instability. The FJO was significantly larger in the DLSS group than in the control group, suggesting that the lumbar segments in DLSS have greater mobility in our study. And the risk of suffering DLSS significantly increased if FJO > 3.75 mm. However, FJA and FJO did not show a relation with other radiographic parameters, and their function in DLSS needs to be further investigated in the next clinical trial.

The atrophy of the multifidus and psoas major muscles was more severe in the DLSS group. Xia et al. [22] found that the atrophy of paravertebral muscles is associated with the severity of stenosis, which is attributed to the denervation of paravertebral muscles after nerve injury or the influence of inflammation and immune response of DD. The point was also confirmed by a basic experiment. Hodges et al. [23] destroyed the disk and nerve roots of mice, then the atrophy and adipocyte clustering appear in multifidus muscle 3 days after the operation, while the contralateral side just shows adipocyte aggregation. Moreover, there was a relation between IDH and the area of multifidus and psoas muscles. A literature pointed out that decreased stability reflex of multifidus muscles will attribute to the desensitization of mechanical receptors caused by the relaxation of viscoelastic tissue within the disk (narrowing of the disk and formation of asymmetric geometry) [24]. The IDH loss caused the spine canal, lateral recess, and intervertebral foramen to narrow. As a result, nerve roots are compressed and the afferent and efferent pathways of the stability reflex are damaged; eventually, the atrophy of muscles happened [23].

CEF was more severe in the DLSS group than in the control group in all lumbar segments in this paper. Rajasekaran et al. [5] thought CEF might be the initial factor for DD. Beth et al. [25] reported that the transport of small molecules would reduce after CEP was damaged and DD begins subsequently. Uruj et al. [26] found DD was associated with the area of endplate damage by autopsy and μCT examination. And higher disk height allows the greater activity of the intervertebral space, and more stress is applied to CEP [16]. As a result, the progress of DLSS was accelerated by CEF by promoting DD. However, the negative linear relation between CEF and DD was only observed at L1-2, L2-3, and L5-S1, which is attributed to the fact that the intervertebral spaces of L3-4 and L4-5 have greater mobility and are subjected to greater stress [18]. Endplate degeneration or defects results in a range of clinical symptoms and diseases by weakening the transport of nutrients and changing the local or overall stress state of the disk [27]. However, the Modic changes showed no difference between the two groups, indicating that Modic changes might not be associated with DLSS.

Our study found that LF was significantly thicker in the DLSS group than in the control group. Sakamaki et al. [28] pointed out that LF would be thicker at all spine segments if the thickness of LF is larger than 3 mm at L2-3. And the thickness of LF at L2-3 was 4.2 ± 0.5 mm in the DLSS group, which was consistent with the previous study. It might be the reason why the patients are vulnerable to symptoms of nerve compression. Peng et al. [11] confirmed that the thickening of LF is associated with stress while LF had no association with ROM in our study, indicating that the thickness of LF is more relevant with lateral and rotational movement. Yabe et al. [29] found the thickness of LF showed a significant relation with age and segments instead of IDH, which is consistent with the results of this study.

## Conclusion

Larger initial disk height, DD, CEF, LF thickening, sagittalized lumbar facet joints, greater facet joint spaces, and atrophied paravertebral muscles were considered to be the risk factors for DLSS. Larger IDH could contribute to DLSS by increasing intervertebral mobility to promote DD and atrophied paravertebral muscles by weakening the stability of the lumbar spine. This study focused on IDH to explore the risk factors and intrinsic mechanism for DLSS, but anatomical, biomechanical studies and multicenter-prospective clinical trials are needed to validate the findings.

## Data Availability

The datasets used or analyzed during the current study are available from the corresponding author on reasonable request.

## Abbreviations

DLSS: Degenerative lumbar spinal stenosis
DD: Disk degeneration
IDH: Intervertebral disk height
CEP: Cartilaginous endplate
CEF: Cartilaginous endplate failure
FJO: Facet joint opening
FJA: Facet joint angle
SCSA: Standard cross-sectional area
IDH_L4-5/L3-4_: The ratio of the IDH of L4-5 and L3-4
iIDH_L4-5_: The initial IDH of L4-5
